# Multi-Scale Interactive Network with Color Attention for Low-Light Image Enhancement

**DOI:** 10.3390/s26010083

**Published:** 2025-12-22

**Authors:** Haoxiang Lu, Changna Qian, Ziming Wang, Zhenbing Liu

**Affiliations:** 1 Guangdong Cardiovascular Institute, Guangdong Provincial People’s Hospital, Guangdong Academy of Sciences, Guangzhou 510080, China; 2 Department of Radiology, Guangdong Provincial People’s Hospital (Guangdong Academy of Medical Sciences), Southern Medical University, Guangzhou 510080, China; 3 Guangdong Provincial Key Laboratory of Artificial Intelligence in Medical Image Analysis and Application, Guangzhou 510080, China; 4 School of Computer and Information Security, Guilin University of Electronic Technology, Guilin 541004, China

**Keywords:** low-light image enhancement, multi-scale feature, transformer, color attention mechanism

## Abstract

Enhancing low-light images is crucial in computer vision applications. Most existing learning-based models often struggle to balance light enhancement and color correction, while images typically contain different types of information at different levels. Hence, we proposed a multi-scale interactive network with color attention named MSINet to effectively explore these different types of information for lowlight image enhancement (LLIE) tasks. Specifically, the MSINet first employs the CNN-based branch built upon stacked residual channel attention blocks (RCABs) to fully explore the image local features. Meanwhile, the Transformer-based branch constructed by Transformer blocks contains cross-scale attention (CSA) and multi-head self-attention (MHSA) to mine the global features. Notably, the local and global features extracted by each RCAB and Transformer block are interacted with by the fusion module. Additionally, the color correction branch (CCB) based upon self-attention (SA) can learn the color distribution information from the lowlight input for further guaranteeing the color fidelity of the final output. Extensive experiments have demonstrated that our proposed MSINet outperforms state-of-the-art LLIE methods in light enhancement and color correction.

## 1. Introduction

Images captured under low-light environments typically suffer from low brightness, low signal-to-noise ratio, and color distortion, which can significantly reduce the performance of high-level vision tasks such as object detection, segmentation, and scene understanding [1,2]. In the early stage, charge-coupled devices (CCD), complementary metal-oxide-semiconductor (CMOS) sensors and other advanced specialised-hardwares are used to obtain high-quality images under suboptimal lighting conditions [3]. But these hardwares are expensive and have operational complexity, limiting their application in the realworld. As a result, researchers gradually focus on designing software-driven low-light image enhancement (LLIE) approaches to restore degraded images with low illumination [4].

Numerous traditional LLIE methods containing histogram equalization (HE) [5], Retinex-based methods [6], and domain transformation-based methods [7] have been proposed for promoting the brightness and contrast of lowlight images. HE-based methods directly adjust the image pixels in a pixel-to-pixel manner to improve the contrast and illumination of lowlight images, but they may lead to over-enhancement and detail loss. Retinex-based methods typically decompose images into illumination and reflectance components to enhance the images’ visibility depending on the specific guidelines. For example, single-scale Retinex (SSR) and multi-scale Retinex with color restoration (MSRCR) [8] try to analyze lowlight images. They tend to introduce noticeable color deviations and blurry detail in enhanced images due to inaccurate estimation of illumination and reflectance components. The domain transformation-based methods transfer the original image into gradient, wavelet domains for detail enhancements, but suffer from a dramatic drop in restoration performance under complexity scenarios.

With the advancement of computing resources, learning-based methods [2,4,9], such as convolutional neural networks (CNNs), generative adversarial networks (GANs), and diffusion model, have proven effective in various computer vision tasks, with the help of their powerful feature extraction and representation capability. LLNet [10], the most groundbreaking learning-based LLIE work, stacked sparse denoising autoencoders for light improvement and denoising simultaneously. In recent years, researchers have proposed many advanced data-driven methods, such as JED [11], SID [12], IPT [13], PairLIE [14], etc., to entirely utilize the inherent relations among low-/normal-illumination images in training datasets, and these LLIE models exhibit outstanding performance in generating visually pleasing results with clearer details. Most above-listed LLIE models gain improvement by injecting the image pyramid [15], Retinex theory [16], meta-learning strategy [9], and other advanced technologies or substantially increasing the depth of the network. However, these LLIE methods seldom utilize the correlation and complementarity of global and local features. In addition, most existing methods only focus on enhancing brightness and contrast, while ignoring the color distribution of the original image.

To address these challenges, this paper proposes a multi-scale interactive network with color attention for lighting up degraded images captured under suboptimal lighting conditions, named MSINet. The proposed MSINet can simultaneously capture local textures and global context to effectively enhance the brightness and details of the inputs as well as remove their color deviation. Our MSINet contains three parallel branches, i.e., CNN-based branch, Transformer-based branch, and Color correction branch. The former can extract local features from the original input by stacking CNN blocks, and the Transformer-based branch based on stacked cross-scale Transformer blocks, including multi-head self-attention (MHSA) and cross-scale attention (CSA), can dig up global features. Meanwhile, the fusion block is introduced to realize the interaction of global and local features for analyzing their correlation and complementarity. The color correction branch built upon the self-attention can fully explore the color distribution in the original images.

We emphasize the primary contributions of this work as follows.

(1) We propose an efficient and robust LLIE method named MSINet, which integrates CNN and Transformer structures for balancing local detail extraction and global feature encoding. Extensive experiments show our MSINet can generate visually pleasing images.
(2) We proposed a cross-scale Transformer module combining cross-scale attention (CSA) and multi-head self-attention (MHSA) to enhance the model’s multi-scale feature learning. Meanwhile, the CNN-based branch can fully explore the local feature.
(3) We proposed a self-attention-based color correction branch to dig up color distribution weighting for color correction in the LLIE tasks. Additionally, we design a fusion block to analyze the correlation and complementarity of global–local features.

We demonstrate the organization of the remainder of this paper as follows. The previous LLIE methods related to our MSINet are reviewed in Section 2. In Section 3, the architecture of our model is demonstrated. In Section 4, we evaluate the proposed method on public benchmarks. Additionally, the analysis of detail enhancement, computational complexity, ablation study, and applications as well as limitations and future work are presented. Finally, the conclusions are given.

## 2. Related Works

### 2.1. CNN-Based LLIE Enhancement

CNN-based LLIE approaches [17,18] have demonstrated dramatic improvements due to their powerful nonlinear representation ability, which can learn nonlinear mapping from the lowlight inputs to their corresponding normal-light versions. For example, Liu et al. [7] designed a Guided Filter-inspired Network (GFNet) for lighting up low-light RAW image enhancement in a guided filter (GF)-like manner. Lim et al. [19] applied the Laplacian pyramid in a multi-scale network to adjust global illumination and restore fine details. Zhang et al. [20] proposed a deep color-consistent network to enhance the naturalness of the image by preserving color information. Although the above-listed methods can light up low-illumination images, they suffer from poor interpretability. Hence, the physical model including Retinex, dehazing model, is used to enhance the interpretability of the LLIE models [17,21]. Wei et al. [18] first injected the Retinex theory into the traditional convolutional neural networks (CNNs) to develop an end-to-end architecture called RetinexNet. Subsuquently, URetinex-Net [16], including initialization, unfolding optimization, and illumination adjustment modules as well as CRetinex [22] decomposing an image into reflectance, color shift, and illumination, is proposed for the LLIE tasks. Recently, multi-scale feature fusion [23,24], perceptual fidelity estimation techniques [25], meta-learning [9], collaborative learning [26], and other advanced technologies are employed to design more efficient LLIE methods. But most of these existing LLIE methods heavily rely on the high-quality paired datasets, limiting their applicability in the real world. To address this challenge, Jiang et al. [27] developed an unsupervised method based on GANs, which integrates a global–local discriminator to improve performance. Guo et al. [28] used a lightweight network that learns enhancement curves and iteratively enhances images. Yao et al. [29] presented a gradient-aware and contrastive-adaptive (GACA) learning framework by estimating more accurate gradient information and introducing a regularization constraint. Yan et al. [30] proposed a Horizontal/Vertical-Intensity (HVI) color space based on the polarized HS maps and learnable intensity. These self-/un-supervised learning-based methods inevitably generate color deviation and blurry details in enhanced images.

### 2.2. Transformer-Based LLIE Methods

The vision transformer (ViT) model based on the self-attention mechanism demonstrates powerful potential in capturing the global dependency of the input feature [31,32]. Cai et al. [33] proposed a one-stage Retinex-based transformer to estimate the illumination information for lighting up the low-light image. Zhang et al. [34] injected the multiple heads into a single network to perform denoising, luminance adjustment, refinement, and detail enhancement for the LLIE. Wu et al. [35] presented two key innovations: an improved Gaussian filtering-based image enhancement module and a hierarchical feature extraction network. Dang et al. [36] employed a lightweight model PPformer to extract both local and non-local information, and further fused them by the dual cross-attention mechanism for the LLIE tasks. Pei et al. [37] proposed a fast Fourier transform embedded noise-aware CNN-Transformer, which removes noise in both the spatial and frequency domains. Brateanu et al. [38] proposed a lightweight transformer-based network containing Channel-Wise Denoiser (CWD), Multi-Stage Squeeze and Excite Fusion (MSEF), and Multi-Headed Self-Attention (MHSA) for promoting the quality of low-light images. Wen et al. [39] performed the pure CNN-based estimator to generate a light-up feature map and a lit-up image, and the restorer based on the U-shaped network equipped with an Illumination-Guided Dual Attention Block (IGDAB) was used to denoise the lit-up image. Dong et al. [40] presented a new multi-scale CNN-Transformer hybrid framework guided by structure priors. Notably, the illumination-invariant edge detectors based on the UNet encoder–decoder architecture with the CNN-Transformer hybrid structure were used to extract robust structure priors. Most ViT-based methods focus on channel modeling to reduce expensive computational costs; however, they introduce spatial illumination inconsistencies, artifacts, and blurry details in restored images.

### 2.3. Diffusion-Based LLIE Methods

The diffusion model is a generative framework that gradually adds noise to disrupt the data structure and then learns the reverse denoising process, which has been wildly used in image restoration [41,42], medical image processing [43], and so on. Yi et al. [44] formulated the low-light image enhancement problem into Retinex decomposition and conditional image generation. Subsequently, they proposed a LLIE method named Diff-Retinex++ containing the Denoising Diffusion Model (DDM) and the Retinex-Driven Mixture of Experts Model (RMoE) [45]. Lin et al. [46] proposed the Attribute Guidance Diffusion framework (AGLLDiff), a training-free method for effective real-world LLIE. Yang et al. [47] employed the Diffusion-guided Degradation Calibration (DDC) module to narrow the gap between real-world and training low-light degradation, further developing the Fine-grained Target domain Distillation (FTD) module to find a more visual-friendly solution space. Huang et al. [48] integrated a size-agnostic diffusion process with a reverse process reconstruction loss to more accurately recover fine details. Hu et al. [49] employed the conditional correlation module (CCM) to effectively integrate color and illumination priors, and the residual decomposition network (RDN) was introduced to generate the reflectance image representing the color object. Jiang et al. [50] injected the wavelet transformation into the conditional diffusion model to achieve stable denoising and reduce randomness during inference. Jin et al. [51] proposed Dual-Conditional Guidance Sparse Diffusion (DCGSD), a physically explainable and prior guidance model, to light up low-illumination images. Although diffusion-based LLIE approaches can generate visually pleasing images from the lowlight inputs, they suffer from time-consuming, excessive computational resource consumption, and unstable restoration.

## 3. Methodology

**Figure 1:**
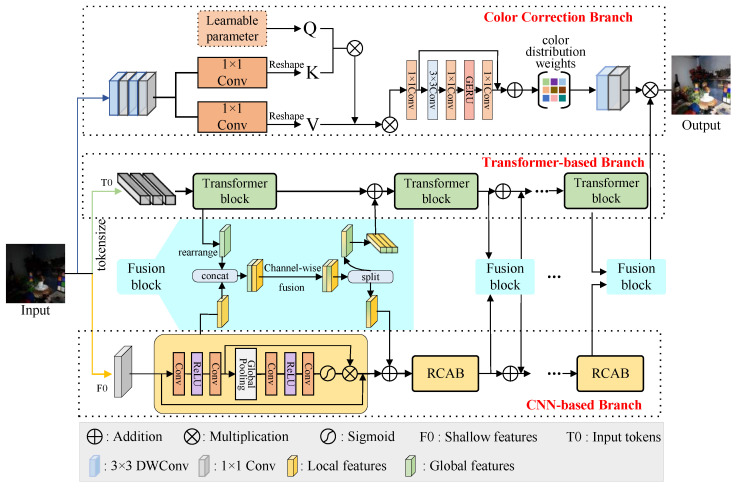
Overview of the proposed model (MSINet), which includes three parrallel branches: CNN-based branch, Transformer-based branch, and color correction branch.

We present the motivation for our proposed method in Figure 1; it can effectively enhance low-light images and preserve color fidelity and fine details. The MSINet is composed of three main components: a CNN-based branch, a Transformer-based branch, and a color correction branch. Specifically, the CNN-based branch first extracts shallow features through a convolutional layer with a size of 1×1. Then, to fully explore the local image features, the shallow features is fed to the successive stacked Residual Channel Attention Blocks (RCABs) (pure CNN blocks). This stage can be formulated as

(1) $$F_{L}=H_{\mathrm{CNN}}\left(I_{\mathrm{low}}\right)=H_{\mathrm{RCAB}}\left({\mathrm{Con}}_{1}\left(I_{\mathrm{low}}\right)\right),$$

where $I_{\mathrm{low}}$ is the lowlight images, $H_{\mathrm{CNN}}\left(\cdot \right)$ represents the CNN-based branch, ${\mathrm{Con}}_{1}$ represents the convolutional layer with a size of 1×1, and $H_{\mathrm{RCAB}}\left(\cdot \right)$ represents the pure CNN block.

The Transformer-based branch performs the successive stacked pure Transformer blocks based on cross-scale attention (CSA) and multi-head self-attention (MHSA) on the inputs to their global features. Meanwhile, the global-local features are aggregated by the fusion blocks to explore their complementarity and correlation.

(2) $$\left\{\begin{array}{c} F_{G}=H_{\mathrm{Trans}}\left(I_{\mathrm{low}}\right)\\ F_{\mathrm{Fu}}=H_{\mathrm{FB}}\left(F_{L},F_{G}\right) \end{array}\right.,$$

where $F_{G}$ represents the global features extracted by the Transformer-based branch $H_{\mathrm{Trans}}\left(\cdot \right)$, and $F_{\mathrm{Fu}}$ represents the fused features generated by the fusion block $H_{\mathrm{FB}}\left(\cdot \right)$.

Finally, the color correction branch employs two successive Conv layers with kernel sizes of 3×3 and 1×1 to detect low-level features, and the learnable self-attention mechanism is used, fully exploring color distribution. We further yield visually pleasing images with vivid color by performing color distribution weighting on the fused feature maps.

(3) $$F_{\mathrm{out}}=F_{\mathrm{Fu}}⊗H_{\mathrm{Trans}}\left({\mathrm{Conv}}_{3}\left({\mathrm{Conv}}_{1}\left(I_{\mathrm{low}}\right)\right)\right).$$

where $F_{\mathrm{out}}$ is the final output, ⊗ represents the multiplication operation, and ${\mathrm{Conv}}_{3}$ represents the convolutional layer with a size of 3×3.

### 3.1. CNN-Based Branch

The CNN shows powerful potential local perception ability for lighting up low-illumination images. In our proposed MSINet, we design a pure CNN-based branch by stacking some Residual Channel Attention Blocks (RCABs). Given a lowlight input $I_{\mathrm{low}}$, the convolutional layer with a size of 1×1 is performed to extract the shallow features of local regions. Subsequently, we feed these features into the stacked Residual Channel Attention Blocks (RCABs) to fully dig up the local image features. And the processing procedure can be defined as

(4) $$\left\{\begin{array}{c} F_{\mathrm{SF}}={\mathrm{con}}_{1\times 1}\left(I_{\mathrm{low}}\right)\\ F_{L}^{i}=F_{\mathrm{RCAB}}^{i}\left(F_{L}^{i-1}\right) \end{array},\;1\leq i\leq N\right..$$

where $F_{RCAB}^{i}$ denotes the $i_{th}$ Residual Channel Attention Block (RCAB), and $F_{L}^{i}$ represents the local features generated by the $i_{th}$ Residual Channel Attention Block (RCAB). Notably, each RCAB only contains a series of Conv layers, ReLU, and global pooling, and the skip connection is also introduced into the RCAB to explore the model’s hierarchical feature extraction ability.

### 3.2. Transformer-Based Branch

**Figure 2:**
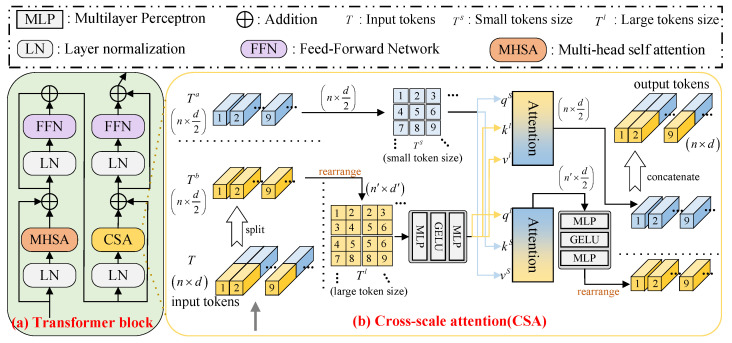
Structure of the Transformer block (**a**) and cross-scale attention (CSA) (**b**).

The Transformer-based branch is designed to analyze the long-range dependency of features for perceiving the global image features. This branch is a pure Transformer dependent upon a stacked transformer block (as illustrated in Figure 2a), and each block contains a cross-scale attention (CSA) module and multi-head self-attention (MSHA) to fully explore the self-correlation and scale correlation of the features. Firstly, the input image $F_{e}\in R^{h\times w\times c}$ is converted into multiple tokens $T_{0}\in R^{n\times d}$, and each token represents a part of the image with dimension *d*. Then, the token $T_{0}\in R^{n\times d}$ is fed into the transformer block, and the operations within each involve several steps, namely

(5) $$\begin{array}{cc} \; & T_{i-1}^{\prime \prime }=\mathrm{FFN}\left(\mathrm{LN}\left(T_{i-1}^{\prime }\right)\right)+T_{i-1}^{\prime },\\ \; & T_{i-1}^{\prime }=\mathrm{MHSA}\left(\mathrm{LN}\left(T_{i-1}\right)\right)+T_{i-1},\\ \; & T_{i}=\mathrm{FFN}\left(\mathrm{LN}\left(T_{i-1}^{\prime \prime \prime }\right)\right)+T_{i-1}^{\prime \prime \prime },\\ \; & T_{i-1}^{\prime \prime \prime }=\mathrm{CSA}\left(\mathrm{LN}\left(T_{i-1}^{\prime \prime }\right)\right)+T_{i-1}^{\prime \prime }. \end{array}$$

where $\mathrm{LN}\left(\cdot \right)$ represents the normalization layer, $\mathrm{FFN}\left(\cdot \right)$ represents the feedforward neural network, and $\mathrm{MHSA}\left(\cdot \right)$ and $\mathrm{CSA}\left(\cdot \right)$ represent the MHSA and CSA, respectively.

***CSA:*** The CSA can promote the model’s ability to represent cross-scale features, and its structure is shown in Figure 2b. We detail a description of the procedure of the CSA as follows: the input tokens are embedded into $T\in R^{m\times d}$, and then split along the axial dimension (i.e., the last dimension) into two parts, $T^{a}\in R^{m\times d/2}$ and $T^{b}\in R^{m\times d/2}$. These parts are then used to generate $T^{\prime }\in R^{m\times d}$, which is subsequently reconstructed to retain the structure of the original token while obtaining larger tokens. In this process, the stride $s^{\prime }$ is closely related to both the number of tokens and their dimensionality.

(6) $$\begin{array}{cc} \; & n^{\prime }=\left[\frac{h-t^{\prime }}{s^{\prime }}+1\right]\times \left[\frac{w-t^{\prime }}{s^{\prime }}+1\right],\\ \; & d^{\prime }=\frac{d\times t^{\prime 2}}{2\times t^{2}}=\left(\frac{c}{2}\right)\times t^{\prime 2}. \end{array}$$

With the help of the CSA, the MSINet can generate a large number of overlapping tokens at different scales, which helps in discovering cross-scale repetitive structures in the image. To better exploit image blocks at different scales and transfer large-scale features to smaller blocks, the model uses tokens of a larger size ($n^{\prime }>n$) during reconstruction. The network then processes $T^{s}$ and $T^{\prime }$ to calculate the cross-scale attention weights, and further extract richer information from these tokens. Specifically, the first step is to generate query, key, and value from the two token sets $T^{s}$ and $T^{\prime }$. And $T^{s}$ corresponds to $\left(q_{s},k_{s},v_{s}\right)\in R^{n\times d/2}$, while $T^{\prime }$ corresponds to $\left(q^{\prime },k^{\prime },v^{\prime }\right)\in R^{n\times d/2}$. Finally, by adjusting the dimensionality, the CSA improves the efficient information transfer between different scales, optimizing the image restoration process without additional computational burden.

As we know, the size of the large and small tokens plays a key role. Hence, we perform the CSA module with different combinations of large and small token sizes on the LOL-v1 dataset. The PSNR scores of our MSINet with different token sizes are presented in Table 1. It can be observed that the small and large token sizes are respectively set to 3 and 4 and can generate higher PSNR scores.

***Fusion module:*** For fully capturing feature representation at different branches, we propose a multi-branch feature fusion module, which horizontally connects the intermediate features of each branch to enable more efficient feature interaction and integration. This design allows the model to leverage the unique strengths of each branch, improving the overall performance of the network. Figure 1 illustrates the specific structure and implementation of the fusion module.

Specifically, for the intermediate features $T_{i}$ and $F_{i}$ output from the $i_{th}$ RCAB and Transformer block, feature fusion is performed through the fusion module $H_{\mathrm{fuse}}$, which combines these features from different branches by capturing cross-branch dependency. The fusion is mathematically expressed as follows:

(7) $$M_{i}=H_{\mathrm{fise}\;}^{i}\left(\mathrm{rearrange}\left(T_{i}\right)∥F_{i}\right),\;1\leq i\leq N$$

where $M_{i}\in R^{2c\times h\times w}$ represents the fused feature representation, where the ‘rearrange‘ operation indicates a reordering of image features, and the symbol ‖ denotes concatenation along the channel dimension. Additionally, the fusion module $H_{\mathrm{fuse}}$ uses a 1×1 convolutional layer to improve feature fusion along the channel dimension. Except for the final fusion module (i.e., i=N), the fused feature $M_{i}$ is evenly split along the channel dimension into two parts, denoted as $M_{T}^{i}\in R^{c\times h\times w}$ and $M_{F}^{i}\in R^{c\times h\times w}.$

### 3.3. Color Correction Branch

This paper introduces a unique color restoration branch designed to enhance the model’s ability to perceive and correct local color information in images. Specifically, the branch first extracts shallow features from the input image by combining deep convolution layers with standard convolution, and then expands the feature channels to enhance the image’s color representation. These enhanced shallow features are then passed through a specially designed Color Attention module, which uses learned query (Q), key (K), and value (V) to efficiently associate features. The Transformer-based self-attention mechanism in this module comprehensively explores and extracts the color features of the image.

This module effectively captures local color features in the image and performs color correction. Compared to traditional image enhancement methods, it more accurately restores the image’s color details. Specifically, by adjusting the convolution kernels and sizes of each convolution layer, the color restoration branch adapts the image’s color balance, reducing color deviations caused by lighting and noise. Finally, through the combination of multiple convolution and Transformer modules, the branch recovers the color details of the image and enhances its visual quality.

### 3.4. Loss Function

This paper employs $L_{1}$ and visual geometry group (VGG) perceptual loss functions to create our proposed method for enhancing lowlight images and detail enhancement. Among them, $L_{1}$ loss is robust and less sensitive to outliers, which can promote stable and faster convergence of the model by calculating the pixel-wise differences between the predicted image and its ground truth. And it can be expressed as

(8) $$L_{1}=\frac{1}{N}\sum_{i=1}^{N}\left|y_{i}-{\hat{y}}_{i}\right|,$$

where *N* is the total number of samples. *y* and $\hat{y}$ represent the reference and output images, respectively.

VGG loss can measure the difference between the predicted image and its ground truth in deep feature space to make the former exhibit visually satisfactory perception. The VGG loss function $L_{vgg}$ can be defined as

(9) $$L_{vgg}=\Psi \left(I^{\prime }\right)-\Psi \left(\hat{I}\right),$$

where Ψ(·) denotes the pre-trained VGG network. $I^{\prime }$ and $\hat{I}$ denote the feature maps extracted by the pre-trained VGG from the ground truth and enhanced images, respectively.

Finally, the total loss function $L_{Total}$ used in this paper can be defined as

(10) $$L_{Total}=L_{1}+\lambda L_{vgg}.$$

where λ is a weighting factor, empirically finding that the total loss function $L_{Total}$ with $\lambda =1\times 10^{-5}$ can guarantee our method generates visually pleasing images.

## 4. Experimental Results and Analysis

This section first describes the implementation details and experimental settings. Next, we perform comprehensive evaluations on paired and unpaired datasets to verify the effectiveness of our MSINet. Finally, the analysis of detail enhancement, computational complexity, ablation study, and applications as well as limitations and future work is performed.

### 4.1. Implementation Details

We use the MIT-Adobe 5K [52], LOL-v1 [18], and LOL-v2 [53] datasets for verifying the performance in the LLIE tasks. Among them, the MIT-Adobe 5K [52] contains 4500 pairs of low-/normal-light training images and 500 pairs of low-/normal-light testing image pairs, the LOL-v1 [18] contains 485 pairs of low-/normal-light training image pairs and 15 pairs of low-/normal-light testing image pairs, and the LOL-v2 [53] contains 900 pairs of low-/normal-light training images and 100 pairs of low-/normal-light testing image pairs. Note that the training and testing images are resized to the size of 512×512×3. Additionally, we also perform our proposed LLIE model on four unpaired datasets, including DICM, LIME, VV, and MEF, to further test its robustness in real-world applications.

All validation experiments are implemented in the Pytorch framework on an NVIDIA Tesla P100 GPU. We augment the training dataset through rotating by $90^{\circ }$ and horizontal flipping. During training, the ADAM optimizer with β1=0.9, β1=0.9999, and $\varepsilon =10^{-8}$ is used to train the parameters of our model. The initial learning rate is set to $1\times 10^{-3}$ and fine-tuned to $1\times 10^{-4}$ after 50 epochs. A batch size of 8 is applied.

### 4.2. Experimental Settings

Our method is compared with eighteen LLIE methods, including traditional methods, LIME [54], JED [11]; supervised learning-based methods, SID [12], IPT [13], RetinexNet [18], STANet [55], MIRNet [56], DRBN [57], KinD [58], LPNet [15], UFormer [16], PairLIE [14] and RetFormer [33], LightenDiffusion [41], QuadPrior [59], CIDNet [30], and END [60]; and self/un-supervised learning-based methods, RUAS [61], DSLR [19], Zero-DCE [28], URetinex-Net [16]. Notably, these above-listed comparison methods use publicly available source codes with recommended parameters to reproduce the enhanced results.

Except for comparison of visual perception, we also adopt commonly used image quality evaluation metrics to quantitatively assess their performance. The peak signal-to-noise ratio (PSNR), structural similarity index (SSIM), and perceptual image patch similarity (LPIPS) are used to evaluate the MIT-Adobe 5K [52], LOL-v1 [18], and LOL-v2 [53] datasets. While the former two metrics are full-reference evaluation, the LPIPS is no-reference evaluation. Among them, a higher PSNR or SSIM score indicates more realistic restoration results, while a lower LPIPS score suggests a better visual perception. For the DICM, LIME, VV, and MEF datasets, the natural image quality evaluator (NIQE), perceptual index (PI), and no-reference image quality metric (NIQMC) are used to assess the enhanced images. And these three metrics are no-reference evaluation. Among them, a lower NIQE or PI score suggests a better natural-looking and visual perception. A higher NIQMC score indicates a greater amount of image information.

### 4.3. Comprehensive Evaluation on Paired Datasets

The visual evaluation of our MSINet is compared with state-of-the-art LLIE approaches on the 5K, LOL-v1, and LOL-v2 datasets to verify their performance in the LLIE tasks.

**Figure 3:**
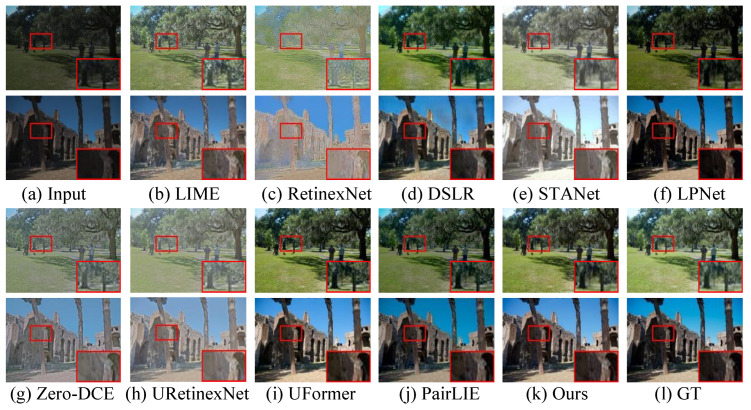
Visual comparisons on the MIT-Adobe 5K dataset. (**a**) The input samples are randomly selected from the MIT-Adobe 5K dataset. The enhanced results generated by (**b**) LIME [54], (**c**) RetinexNet [18], (**d**) DSLR [19], (**e**) STANet [55], (**f**) LPNet [15], (**g**) Zero-DCE [28], (**h**) URetinex-Net [16], (**i**) UFormer [16], (**j**) PairLIE [14], (**k**) ours, and (**l**) Ground truth (GT).

**Qualitative Evaluation.** On the MIT-Adobe 5K dataset, as shown in Figure 3, LIME [54] and RetinexNet [18] struggle with overexposure and unnatural color shifts. And the latter yields unnatural-looking visual experience and blurry details. DSLR [19] creates high-contrast images with observable artifact haloes. STANet [55] introduces local overenhancement and unwanted color deviation. Although LPNet [15] effectively lights up the brightness of lowlight images, it is unsatisfactory in removing local underenhancement. Zero-DCE [28] and URetinex-Net [16] yield hazy-like and visually unnatural-looking images from the lowlight inputs, and they also fail in removing color distortion and inherent noise. UFormer [16] shows satisfactory performance in the LLIE task, but some UFormer-enhanced images exhibit hazy-like (e.g., the second row in Figure 3i) and local dark areas (e.g., the third row in Figure 3i). PairLIE [14] struggles to balance the contrast stretch and detail enhancement for the low-illumination images. In contrast, our proposed method achieves a more balanced enhancement performance, characterized by refined texture rendition and minimized perceptual distortion, thereby yielding superior visual fidelity.

**Figure 4:**
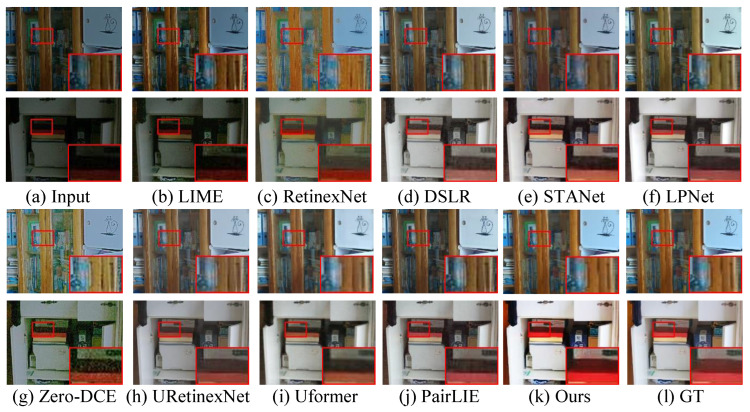
Visual comparisons on the LOL-v1 dataset. (**a**) The input samples are randomly selected from the LOL-v1 dataset. The enhanced results generated by (**b**) LIME [54], (**c**) RetinexNet [18], (**d**) DSLR [19], (**e**) STANet [55], (**f**) LPNet [15], (**g**) Zero-DCE [28], (**h**) URetinex-Net [16], (**i**) UFormer [16], (**j**) PairLIE [14], (**k**) ours, and (**l**) Ground truth (GT).

**Figure 5:**
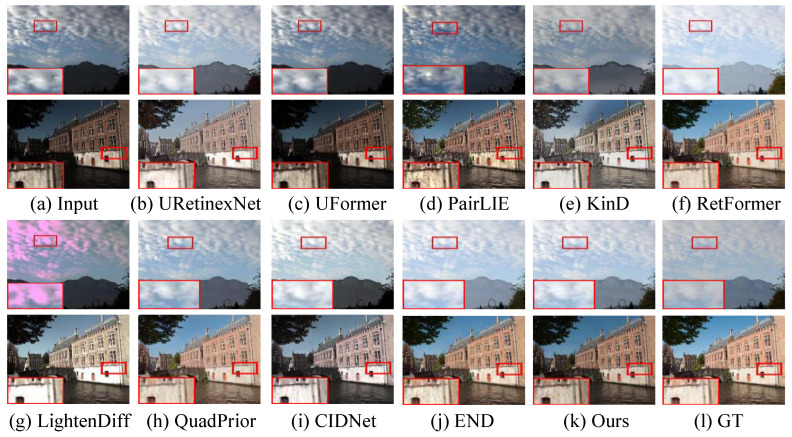
Visual comparisons on the LOL-v2 dataset. (**a**) The input samples are randomly selected from the LOL-v2 dataset. The enhanced results generated by (**b**) LIME [54], (**c**) RetinexNet [18], (**d**) DSLR [19], (**e**) STANet [55], (**f**) LPNet [15], (**g**) Zero-DCE [28], (**h**) URetinex-Net [16], (**i**) UFormer [16], (**j**) PairLIE [14], (**k**) ours, and (**l**) Ground truth (GT).

Figure 4 and Figure 5 present the enhanced results randomly selected from the LOL-v1 and LOL-v2 datasets. For the LOL-v1 dataset, RetinexNet [18] and Zero-DCE [28] generate a greenish tone and unsatisfactory visual perception. LIME [54] cannot yield high-contrast images or remove local darkness. DSLR [19], URetinex-Net [16], and LPNet [15] fail to achieve color correction, and the former two methods inject blurry details and edges in enhanced images. STANet [55] and PairLIE [14] promote the quality of lowlight images, but successfully remove artifact haloes. For the LOL-v2 dataset, all comparison methods cannot light up the partially dark areas. Among them, the performance of the UFormer [16] is the poorest, followed by that of PairLIE [14]. URetinex-Net [16] generates high-brightness images with unsatisfactory contrast. LightenDiffusion [41] introduces color deviation (e.g., the sky part of the image in Figure 5g), and QuadPrior [59] fails in removing inherent noise and making details clearer. CIDNet [30] injects observable color deviation and partial darkness. In comparison, our method effectively removes color deviations, highlights the structural details, and improves visibility without over-enhancement or oversaturation.

**Figure 6:**
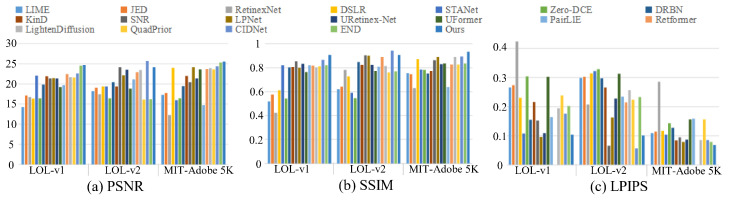
The bar charts of the PSNR, SSIM, and LPIPS of different LLIE methods on paired datasets.

**Quantitative Evaluation.** From the quantitative evaluation scores on the LOL-v1, LOL-v2, and MIT-Adobe 5K datasets in Table 2, it can be seen that our MSINet has higher scores of the PSNR, SSIM, and LPIPS on the MIT-Adobe 5K dataset than the compared methods. On the LOL-v1 dataset, the proposed MSINet has comparable and higher values of the PSNR and SSIM. Additionally, our MSINet achieves the second-best performance on the LOL-v2 dataset. To clearly demonstrate the PSNR, SSIM, and LPIPS scores on the LOL-v1, LOL-v2, and MIT-Adobe 5K datasets, we further drew their bar charts in Figure 6. The qualitative and quantitative results suggest that our MSINet can effectively produce high visibility and natural color with significant enhancement of the contrast, brightness, and texture details.

### 4.4. Comprehensive Evaluation on Unpaired Datasets

We perform them on unpaired datasets, including DICM, LIME, VV, and MEF, to further evaluate the robustness of our MSINet and other compared LLIE methods in both qualitative and quantitative assessments.

**Figure 7:**
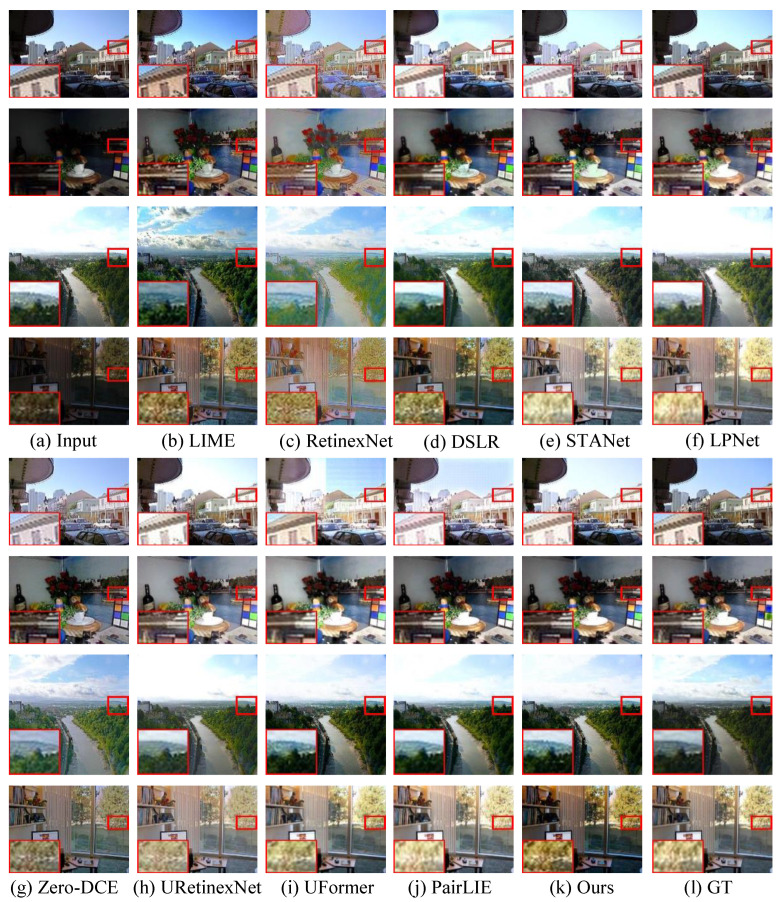
Visual comparisons on paired datasets. From top to bottom, (**a**) the inputs are randomly selected from the DICM, LIME, VV, and MEF, respectively. Their corresponding enhanced results generated by (**b**) LIME [54], (**c**) RetinexNet [18], (**d**) DSLR [19], (**e**) STANet [55], (**f**) LPNet [15], (**g**) Zero-DCE [28], (**h**) URetinex-Net [16], (**i**) UFormer [16], (**j**) PairLIE [14], (**k**) RetFormer [33], and (**l**) ours.

**Qualitative Evaluation.** Figure 7 demonstrates enhanced results generated by these state-of-the-art approaches randomly selected from the DICM, LIME, VV, and MEF benchmarks. The following observations from these enhanced images can be easily obtained: The original lowlight images suffer from local low-contrast and unsatisfactory contrast and illumination as well as blurry details. RetinexNet [18] successfully lights up lowlight images, but generates unnatural-looking visual experience and unwanted artefact haloes. LIME [54] consistently yields visually pleasing results without fine-tuning its parameters on these four datasets. But the LIME-enhanced images still confront lower contrast and color deviation. DSLR [19] inevitably introduces blocking effects in enhanced results (e.g., the third/forth rows in Figure 7d) and fails in removing local extremely lowlight areas. URetinex-Net [16] and UFommer [31] show unsatisfactory performance in removing local over-enhancement. In addition, UFommer [31] introduces an observable checkerboard effect in some enhanced images. Zero-DCE [28] can effectively remove unwanted local extremely-low-illumination areas, while it fails in detail enhancement and noise suppression. RetFormer [33], UFormer [16], DSLR [19], PairLIE [14], and STANet [55] cannot tackle local under-exposure and fail in local detail boosting. STANet [55] introduces color distortion (e.g., the second/third rows in Figure 7e) and local over-enhancement (e.g., the third row in Figure 7e) for some low-illumination images. PairLIE [14] can yield observable artefact haloes, as demonstrated in the first row in Figure 7j. RetFormer [33] also fails in removing color distortion. LPNet [15] generates high-contrast images and clearer details, while unsuccessfully removing local over-enhancement (e.g., the third row in Figure 7f) and enhancing the contrast of the dark areas. In comparison, our model produces visually appealing results with better control over exposure, preserving the details in both bright and dark regions. The image sharpness and clarity are enhanced while maintaining a natural appearance. The fine textures, especially in the foreground and background, are well-preserved, and there are no noticeable color shifts or over-enhancement.

**Figure 8:**
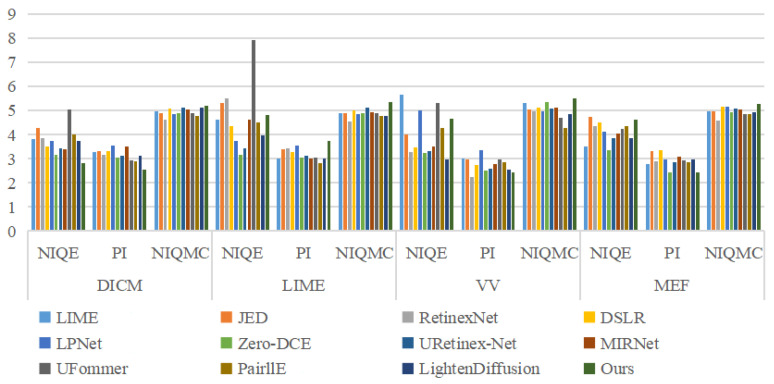
The bar charts of the NIQE, PI, and NIQMC of different LLIE methods on unpaired datasets.

**Quantitative Evaluation.** We further employ the NIQE, PI, and NIQMC to evaluate the performance of these LLIE methods quantitatively. The NIQE, PI, and NIQMC scores of different LLIE methods on the DICM, LIME, VV, and MEF datasets are shown in Table 3. From the quantitative evaluation scores in Table 3, it can be found that our MSINet achieves the best scores in NIQE (2.816), PI (2.553), and NIQMC (5.219) on the DICM dataset. On other datasets, our method also yields lower NIQMC and the second-best PI scores than the compared LLIE methods. For clearly demonstrating the NIQE, PI, and NIQMC scores on the DICM, LIME, VV, and MEF datasets, we further drew their bar charts in Figure 8. In conclusion, the qualitative and quantitative results suggest that our MSINet works better in color correction, detail boosting, and contrast stretch for the LLIE tasks.

### 4.5. Comprehensive Evaluation of Detail Enhancement

High-quality images with clearer details play an important role in object detection and scene understanding. We compare our proposed MSINet with other compared LLIE in terms of detail boosting. As illustrated in Figure 3, Figure 4, Figure 5, Figure 6 and Figure 7, our proposed MSINet significantly lights up lowlight images and removes color deviation from a global view. From a local view, our method can effectively make the fine structural details clearer than other compared methods. Furthermore, we introduce the average gradient (AG), local variance (LVar) and local standard deviation (LSTD) to verify the detail enhancement capability of the model. Among them, the AG can measure the detail information, while the LVar and LSTD can measure the spatial variations in noise and features. Table 4 presents the AG, LVar, and LSTD scores of different LLIE methods. From Table 4, it can be easily found that our proposed MSINet yields more satisfactory AG, LVar, and LSTD scores than the compared approaches. The quantitative and qualitative results indicate that our MSINet is superior compared to LLIE methods in detail enhancement.

### 4.6. Comprehensive Evaluation of Color Correction

Most LLIE methods can light up images and enhance details, but inevitably introduce color deviations. In our experiments, we further verify the color correction ability of our MSINet on the LOL-v1 dataset. Additionally, the ΔE [7] and Bhattacharyya Distance (BD) [63] are introduced to quantitatively evaluate the color correction capability of the compared LLIE methods and our MSINet. Among them, the former is a metric for color distortion, and the latter can measure the color difference between the enhanced images and their reference ones. Table 4 presents the average ΔE and Bhattacharyya Distance (BD) scores of different LLIE methods. It can be easily observed that our method yields lower BD and ΔE scores than the compared methods, indicating that the enhanced images generated by the MSINet share similar color distribution with the reference image.

**Figure 9:**
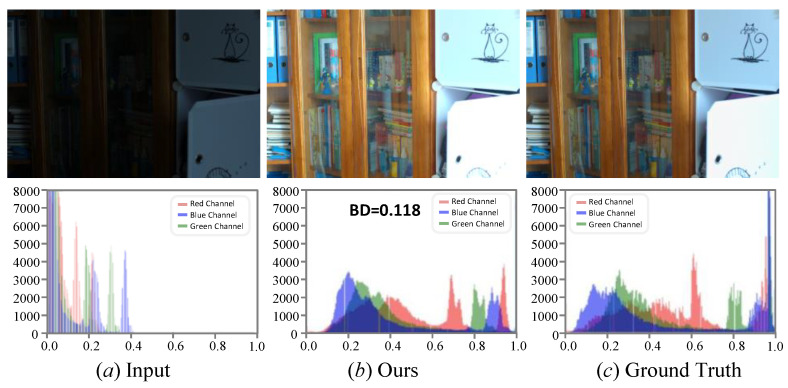
Visual and corresponding histogram comparisons of the original, enhanced, and reference images.

Following references [64,65], we present the visual and corresponding histogram of the original, enhanced, and reference images (as shown in Figure 9) to further demonstrate the physical process of our MSINet for the LLIE tasks. From Figure 9, we can observe that the original image exhibits low illumination and color deviation, and its corresponding histogram is distributed in the area with smaller pixel values. The image processed by our MSINet shows high brightness and vivid color, and its corresponding histogram is distributed more uniformly by pixel interpolation for the lowlight input. The most likely reason is that the Bayer filter sensors make only the smaller value pixels receive the wavelength light under suboptimal lighting conditions. Our proposed MSINet reduces the difference in spectral response among the wavelengths of the R, G, and B channels for removing the color deviation from the enhanced results.

### 4.7. Comprehensive Evaluation of Computational Complexity

Table 5 presents the Param, Flops, and runtime of our MSINet and all the above-listed comparison LLIE methods on the LOL-v1 dataset to comprehensively evaluate their computational complexity. It can be easily found that Zero-DCE [28] enjoys lower computational complexity and faster inference speed. LightenDiffusion [41] encounters a heavy computational burden, limiting its application in the real-world. In contrast, our proposed MSINet can balance the computational complexity as well as inference speed and enhanced results for the LLIE tasks.

### 4.8. Ablation Study

We further perform an ablation study on our proposed MSINet to test the effectiveness of each component. The ablation studies include (a) our method without residual channel Attention block (-w/o RCAB), (b) our method without Cross-Scale Attention Module (-w/o CSA), and our method without color correction Branch (-w/o CCB).

**Figure 10:**
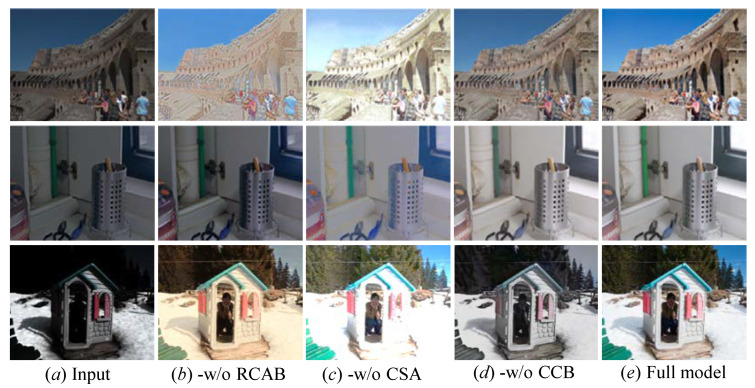
Qualitative ablation results for each key component of our method on paired LOL-v2 datasets. (**a**) Raw images. (**b**) -w/o RCAB. (**c**) -w/o CSA. (**d**) -w/o CCB. (**e**) MSINet (full model).

Figure 10 shows the visual comparisons on the MIT-Adobe 5K, LOL-v1, and LOL-v2 datasets. The following visual results can be observed: (1) -w/o RCAB fails in detail boosting for the LLIE tasks and introduces unnatural-looking visual experience in some enhanced images; (2) -w/o CSA lights up low-illumination images and promotes contrast, but the enhanced images suffer from blurry boundaries and details; (3) -w/o CCB generates observed color deviation in enhanced images; (4) the full model (MSINet) with all key components can effectively yield visually pleasing images with vivid color and clearer details.

Furthermore, we present the quantitative scores of the ablated models for the MIT-Adobe 5K, LOL-v1, and LOL-v2 datasets in Table 6. It can be found that our MSINet can create more satisfactory quantitative scores than the ablated models across three public benchmarks, benefiting from each key component.

### 4.9. Generalization of Our Proposed Method

**Figure 11:**
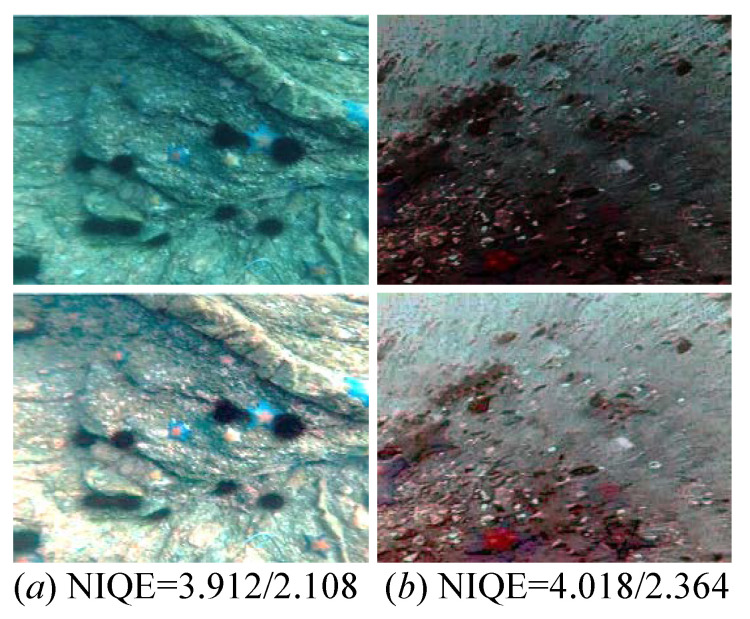
Samples of our enhanced results (bottom) were randomly selected from the UCCS benchmark. -/- suggests the NIQE score of the input and its corresponding enhanced images.

We first perform our method on underwater images to test the generalization of our proposed method. The training/testing datasets are randomly selected from the UCCS benchmark in the ratio of 7:3. Figure 11 demonstrates the enhanced results randomly selected from the UCCS benchmark. Intuitively, the input underwater images exhibit unsatisfactory visual perception, whereas our method can effectively promote the quality of the underwater images. We also present the NIQE score of the input and its corresponding enhanced images, and the latter has a superior NIQE score.

**Figure 12:**
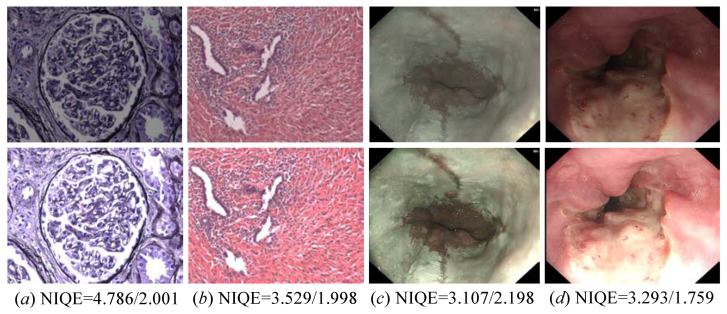
Visual results of our proposed method for enhancing pathological images (the former two columns) and endoscopic images (the latter two columns) with low contrast. From top to bottom, the original images as well as their corresponding enhanced images yielded by our MSINet. -/- suggests the NIQE score of the original input and its corresponding enhanced results.

The proposed MSINet also works well on medical images including endoscopic and pathological images with low contrast. Notably, the endoscopic images are randomly selected from the CVC-ClinicDB dataset (https://tianchi.aliyun.com/dataset/93690 (accessed on 20 October 2025)), and the pathological images are randomly selected from the LUAD-HistoSeg dataset (https://drive.google.com/drive/folders/1E3Yei3Or3xJXukHIybZAgochxfn6FJpr (accessed on 20 October 2025)). As illustrated in the first rows of Figure 12, the original endoscopic images and pathological images encounter low contrast and blurry details, which may deliver compromised information for disease diagnosis, prognosis analysis, and therapeutic effect prediction in clinical analysis. On the contrary, our MSINet shows satisfactory performance in contrast stretch and detail boosting for endoscopic images and pathological images. In addition, we also present the NIQE score of the original input and its corresponding enhanced results. It can be easily found that the enhanced results generated by the MSINet can generate more satisfactory NIQE scores. Experiments suggest that our MSINet exhibits solid generalization in restoring the quality of the endoscopic images and pathological images.

### 4.10. Limitations and Future Work

**Figure 13:**
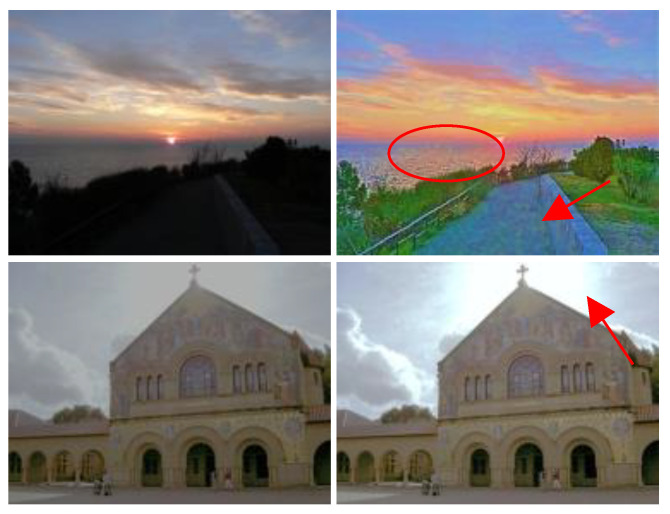
Visual results of our proposed method for some failure instances. From left to right, the original images as well as their correspongding enhanced image yielded by our method. The circle and arrow are used to indicate the areas where image enhancement fails.

The LLIE method can enhance lowlight images and further promote the performance of object detection, image classification, and other advanced computer vision tasks. Our proposed MSINet can work well in yielding more satisfactory results with vivid color and clearer details from low-illumination images in most situations, whereas it fails in noise reduction, local exposure control for lowlight images with local over-exposure, boosted noise, and so on. For example, Figure 13 illustrates some failure instances created by our proposed MSINet. Intuitively, the enhanced images exhibit vivid color and high contrast, but they also contain observed noise, artefact halos, and local over-enhancement. The reason may be that our MSINet directly processes high-frequency components with the inherent noise of the lowlight input. Additionally, our method does not take into account the enhancement of images with local overexposure. In the future, we will promote learning and a specialised denoising module for tackling these challenging issues.

## 5. Conclusions

This paper presents a multi-scale interactive network with color attention named MSINet for light enhancement and color correction. The MSINet is a three-branch CNN-Transformer hybrid structure containing CNN-based branch, Transformer-based branch, and a color correction branch. The CNN-based branch, a pure CNN network, can fully explore the local image features by the stacked residual channel attention blocks (RCABs), and the Transformer-based branch, a pure Transformer network, can mine the global features by the stacked Transformer blocks containing cross-scale attention (CSA) and multi-head self-attention (MHSA). Meanwhile, we design a fusion module to integrate the global and local features extracted by each RCAB and Transformer block. Additionally, we further employ the color correction branch based on self-attention (SA) to learn the color distribution information for removing the color deviation. Experimental results on different datasets have shown that our MSINet can generate visually pleasing images with clearer details and more vivid color compared with LLIE methods.

## Figures and Tables

**Table 1:** The PSNR score of the CSA with different combinations of large and small token sizes on the LOL-v1 dataset. Bold text means the best performance.

Token Sizes	1	2	3	4	5
1	19.88	20.16	20.73	20.89	21.03
2	20.16	22.12	22.75	22.83	22.96
3	20.73	22.75	23.03	**24.72**	24.62
4	20.89	22.83	**24.72**	24.61	24.32
5	21.03	22.96	24.62	24.32	24.27
6	22.26	23.41	24.10	24.28	24.19

**Table 2:** Quantitative analysis of different comparison methods on paired benchmarks. Red/blue text means the best/second-best performance.

Methods	LOL-v1			LOL-v2			MIT-Adobe 5K		
	PSNR	SSIM	LPIPS	PSNR	SSIM	LPIPS	PSNR	SSIM	LPIPS
LIME [54]	14.26	0.5187	0.2659	18.24	0.6224	0.2987	17.31	0.7567	0.1089
JED [11]	17.15	0.5773	0.2741	19.05	0.6431	0.3025	17.72	0.7476	0.1139
RetinexNet [18]	16.76	0.4239	0.4239	17.41	0.7811	0.2074	12.30	0.6298	0.2861
DSLR [19]	16.35	0.6129	0.2307	19.37	0.7273	0.3146	24.01	0.8734	0.1164
STANet [55]	22.03	0.8213	0.1073	19.36	0.5911	0.3227	15.98	0.7854	0.1039
Zero-DCE [28]	16.43	0.5421	0.3034	16.43	0.5474	0.3291	16.39	0.7839	0.1435
DRBN [57]	19.86	0.8046	0.1547	20.38	0.8491	0.2977	19.45	0.7521	0.1275
KinD [58]	21.87	0.8077	0.2157	19.36	0.8241	0.2657	21.95	0.7726	0.0833
SNR [62]	21.31	0.8564	0.1529	24.14	0.9028	0.0658	20.44	0.8641	0.0942
LPNet [15]	21.43	0.8019	0.0955	22.09	0.9014	0.1636	24.19	0.8916	0.0793
URetinex-Net [16]	21.33	0.8346	0.1084	23.50	0.8257	0.2282	21.33	0.8296	0.0861
UFormer [31]	19.25	0.7635	0.3029	18.82	0.7745	0.3134	23.64	0.8366	0.1563
PairLlE [14]	19.68	0.8233	0.1637	21.14	0.8107	0.2343	14.69	0.6398	0.1589
Retformer [33]	22.43	0.8183	—	22.94	0.8905	0.2147	23.67	0.8274	—
LightenDiffusion [41]	21.65	0.8047	0.1947	23.48	0.8155	0.2561	23.95	0.8927	0.0846
QuadPrior [59]	21.59	0.8142	0.2375	16.10	0.7624	0.2240	23.61	0.8277	0.1564
CIDNet [30]	22.59	0.8671	0.1766	25.70	0.9424	0.0562	24.43	0.8936	0.0846
END [60]	24.57	0.8207	0.2031	16.17	0.7714	0.2323	25.34	0.8361	0.0791
Ours	24.72	0.9078	0.1039	24.14	0.9077	0.1009	25.59	0.9346	0.0681

**Table 3:** Quantitative assessments of different LLIE methods on the DICM, LIME, VV, and MEF datasets. Red/blue text means the best/second-best performance.

Methods	DICM			LIME			VV			MEF		
	NIQE	PI	NIQMC	NIQE	PI	NIQMC	NIQE	PI	NIQMC	NIQE	PI	NIQMC
LIME [54]	3.836	3.264	4.952	4.637	2.994	4.881	5.672	3.023	5.326	3.499	2.778	4.979
JED [11]	4.287	3.319	4.893	5.304	3.384	4.895	4.014	2.982	5.035	4.741	3.309	4.951
RetinexNet [18]	3.862	3.181	4.634	5.518	3.427	4.531	3.278	2.244	4.959	4.355	2.902	4.572
DSLR [19]	3.513	3.326	5.097	4.372	3.262	5.015	3.4626	2.753	5.138	4.492	3.342	5.175
LPNet [15]	3.752	3.533	4.841	3.752	3.533	4.841	4.996	3.359	4.977	4.116	2.958	5.144
Zero-DCE [28]	3.169	3.055	4.896	3.169	3.055	4.896	3.261	2.501	5.370	3.369	2.429	4.943
URetinex-Net [16]	3.425	3.146	5.113	3.425	3.146	5.113	3.323	2.573	5.094	3.841	2.858	5.083
MIRNet [56]	3.384	3.528	5.061	4.625	3.028	4.937	3.513	2.774	5.106	4.045	3.097	5.036
UFommer [31]	5.054	2.954	4.885	7.927	3.061	4.893	5.308	2.963	4.687	4.258	2.946	4.857
PairllE [14]	4.016	2.893	4.791	4.519	2.813	4.773	4.269	2.846	4.293	4.337	2.861	4.839
LightenDiffusion [41]	3.741	3.132	5.106	3.968	3.016	4.762	2.968	2.553	4.837	3.843	2.961	4.927
Ours	2.816	2.553	5.219	4.826	3.758	5.364	4.681	2.438	5.522	4.637	2.439	5.274

**Table 4:** Quantitative comparison of existing LLIE methods on the LOL-v1 dataset. Bold text means the best performance.

Method	AG	LVar	LSTD	ΔE	BD
RetinexNet [18]	5.149	3.101	1.062	7.342	0.273
KinD [58]	6.123	2.342	1.002	6.206	0.231
END [60]	6.554	2.657	2.374	5.110	0.159
Zero-DCE [28]	5.221	1.697	1.010	7.091	0.139
SNR [62]	6.335	2.997	1.892	2.310	0.173
CIDNet [30]	6.687	3.028	0.994	3.740	0.211
LightenDiffusion [41]	5.908	5.017	1.930	5.107	0.193
EnlightenGAN [27]	5.712	1.101	2.371	4.529	0.123
PairLIE [14]	5.826	2.017	1.909	4.937	0.187
DSLR [19]	5.290	4.007	2.062	4.387	0.208
UFormer [31]	5.250	3.394	2.107	4.320	0.118
URetinex-Net [31]	5.937	1.997	4.004	7.863	0.211
Ours	**7.358**	**1.075**	**0.937**	**3.871**	**0.109**

**Table 5:** Computational complexity comparison of existing LLIE methods on the LOL-v1 dataset. Bold text means the best performance.

Method	Param (M)	Flops (G)	Time (s)
RetinexNet [18]	1.23	6.79	0.5217
KinD [58]	8.49	7.44	0.6445
END [60]	8.36	270.42	0.7963
Zero-DCE [28]	1.21	5.21	0.0079
SNR [62]	4.01	26.35	0.5141
CIDNet [30]	1.98	8.03	0.7869
LightenDiffusion [41]	101.71	210G	1.2001
EnlightenGAN [27]	8.64	7.88	0.6501
PairLIE [14]	2.16	19.24	0.2971
DSLR [19]	14.31	22.95	0.9210
UFormer [31]	5.20	10.68	0.6298
URetinex-Net [31]	26.27	90.61	0.8902
Ours	**1.99**	**8.24**	**0.1009**

**Table 6:** Ablation study on the MIT-Adobe 5K, LOL-v1, and LOL-v2 datasets. Red/blue text means the best/second-best performance.

Ablated Model	MIT-Adobe 5K			LOL-v1			LOL-v2		
	PSNR	SSIM	LPIPS	PSNR	SSIM	LPIPS	PSNR	SSIM	LPIPS
-w/o RCAB	21.30	0.738	0.071	20.81	0.793	0.187	23.12	0. 812	0.167
-w/o CSA	22.52	0.804	0.089	23.19	0.806	0.174	23.91	0.878	0.157
-w/o CCB	23.09	0.831	0.106	22.14	0.855	0.187	23.68	0.893	0.169
full model	25.59	0.934	0.067	24.72	0.903	0.102	24.14	0.908	0.101

## Data Availability

Publicly available datasets were analyzed in this study. These data can be found here: LOL-v1 benchmark https://daooshee.github.io/BMVC2018website/ (accessed on 1 October 2025), LOL-v2 benchmark https://www.kaggle.com/datasets/tanhyml/lol-v2-dataset (accessed on 1 October 2025), MIT-Adobe 5K benchmark https://data.csail.mit.edu/graphics/fivek/ (accessed on 1 October 2025), and LIME, MEF, DICM, and VV https://drive.google.com/drive/folders/1lp6m5JE3kf3M66Dicbx5wSnvhxt90V4T (accessed on 1 October 2025).
